# Apport de l'imagerie dans le diagnostic des sacroiliites infectieuses : à propos de 19 cas

**DOI:** 10.11604/pamj.2014.17.171.2716

**Published:** 2014-03-06

**Authors:** Hanen Abid, Salim Chaabouni, Faten Frikha, Nozha Toumi, Basma Souissi, Dorra Lahiani, Zouhir Bahloul, Khaireddine Ben Mahfoudh

**Affiliations:** 1 Service de Radiologie CHU Habib Bourguiba 3029 Sfax, Tunisie; 2 Service de Médecine interne CHU Hédi Chaker 3029 Sfax, Tunisie; 3 Service de maladies infectieuses CHU Hédi Chaker 3029 Sfax, Tunisie

**Keywords:** Articulation sacro-iliaque, sacroiliite infectieuse, tuberculose, brucellose, tomodensitométrie, IRM, Sacroiliac joint, infectious sacroiliitis, tuberculosis, brucellosis, CT, MRI

## Abstract

Les sacro-iliites infectieuses méritent d’être mieux connues. Leur diagnostic est souvent retardé en raison d'une symptomatologie trompeuse et des diffcultés d'exploration de l'articulation sacro-iliaque. Notre travail est basé sur une étude rétrospective portant sur les cas de SII, recueillis sur une période comprise entre 1997 et 2008 dans notre centre universitaire Sfax-Tunisie. Le diagnostic de sacro-iliite était retenu en présence d'arguments cliniques et radiologiques d'atteinte sacroiliaque. Nous rapportons dix neuf cas de sacroiliites infectieuses (10 hommes et 9 femmes), avec un âge moyen de 32 ans. L'atteinte était unilatérale dans tous les cas. Les radiographies standard faites dans tous les cas ont été suggestives dans 14 cas et normales dans les autres cas. La TDM faite dans 13 cas a montré, un abcès des parties molles dans 8 cas et un séquestre osseux dans 2 cas. L'IRM réalisée dans 8 cas, a objectivé une infiltration des parties molles dans tous les cas et un abcès dans 3 cas. Le germe a été identifié dans 9 cas (3 cas de tuberculose, 3 cas de brucellose, 2 sacro-iliites à pyogène et un cas de candidose). Cette identification était faite par biopsie dans 3 cas, hémocultures dans 2 cas, prélèvement au niveau de la porte d'entrée dans 1 cas et sérodiagnostic dans 3 cas. Pour les autres cas, l'origine pyogène a été retenue sur des arguments cliniques et biologiques. L'imagerie joue un rôle primordial dans le diagnostic précoce et l'orientation étiologique d'une sacroiliite infectieuse.

## Introduction

Les sacroiliites infectieuses (SII) sont rares et ne représentent que 1 à 4% des infections ostéoarticulaires [1, 2, 3, 4]. La situation profonde de l'articulation est à l'origine d'une sémiologie parfois trompeuse (confondue avec une atteinte de la hanche ou de la charnière lombo-sacrée) responsable d'un retard au diagnostic et par conséquent d'un retard thérapeutique qui peuvent atteindre plusieurs semaines.

Les remaniements des articulations sacro-iliaques sont d'interprétation difficile sur les radiographies standards en raison de leur anatomie complexe et difficile. Le recours à des explorations para cliniques est souvent nécessaire.

A travers ce travail et une revue de la littérature, nous essayerons d'analyser les caractéristiques cliniques, bactériologiques et radiologiques et de montrer l'intérêt de l'imagerie en coupes dans le diagnostic précoce, l'orientation vers l'origine infectieuse d'une sacroiliite ainsi que le germe en cause.

## Méthodes

Notre travail est basé sur une étude rétrospective portant sur 19 cas de SII, recueillis sur une période de 12 ans comprise entre 1997 et 2008. Ont été inclus les patients dont le diagnostic de sacro-iliite était retenu en présence d'arguments cliniques et radiologiques d'atteinte sacroiliaque. L'origine infectieuse de la sacro-iliite a été confirmée par l'isolement du germe en cause dans le foyer articulaire ou au sein d'un abcès profond, ou par la présence de signes histologiques compatibles avec le diagnostic de SII. À défaut d'un diagnostic de certitude, le diagnostic était retenu devant des arguments de présomption épidémiologiques, cliniques, biologiques et radiologiques.

Tous les malades ont bénéficié d'un examen clinique et radiologique des articulations sacro-iliaques. Une radiographie simple a été réalisée dans tous les cas. Une tomodensitométrie (TDM) et une imagerie par résonance magnétique (IRM) des sacro-iliaques ont été faites respectivement dans 13 et 8 cas.

L’étude TDM est centrée sur le sacrum en utilisant un filtre osseux et un filtre mou. Les reformatages utilisées sont coronale (perpendiculaire au plateau supérieur de S1) et axiale oblique (parallèle au plateau supérieur de S1). Les séquences pratiquées en IRM sont le STIR (plan axial et coronal), T1 (plan axial et/ou coronal) et T1 après injection de Gadolinium (plan axial et coronal). Des examens biologiques, sérologiques et bactériologiques ont été effectués selon l'orientation étiologique.

## Résultats

Entre 1997 et 2008, dix neuf malades présentant une S.I.I. ont été hospitalisés, l'incidence annuelle moyenne est de 1,6 nouveaux cas par an. L’âge moyen était de 32 ans (extrêmes de 7 à 70 ans). Le sex-ratio était de 10 /9. Le mode d'installation était brutal dans 11 cas et progressif dans 8 cas. Une douleur et une impotence fonctionnelle étaient présentes dans tous les cas. L'atteinte était unilatérale dans tous les cas: droite dans 7 cas et gauche dans 12 cas. Le siège de cette douleur était variable, souvent au niveau de la région fessière (10 cas), et au niveau de la hanche (6 cas), mais aussi en regard du grand trochanter et au niveau de la fosse iliaque droite rapportée dans 3 cas. Une élévation de la température était notée dans 14 cas. Un syndrome inflammatoire biologique était trouvé dans 11 cas. L'hyperleucocytose était notée dans 11 cas et une leucopénie était trouvée dans 4 cas.

Une porte d'entrée infectieuse était retrouvée dans 5 cas, il s'agissait d'une porte d'entrée cutanée dans 3 cas, génitale dans les deux autres cas. Le délai de confirmation diagnostique par rapport à l'hospitalisation dans notre étude était de 30 Jours avec des extrêmes allant de 7 à 45 jours, témoignant de la difficulté diagnostique.

**Figure 1:**
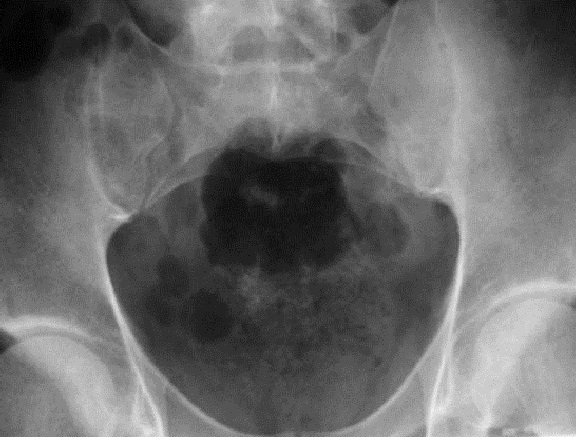
Radiographie standard du bassin de face; Aspect flou condensé des berges de la sacroiliaque gauche; Aspect normal de la sacroiliaque droite

**Figure 2:**
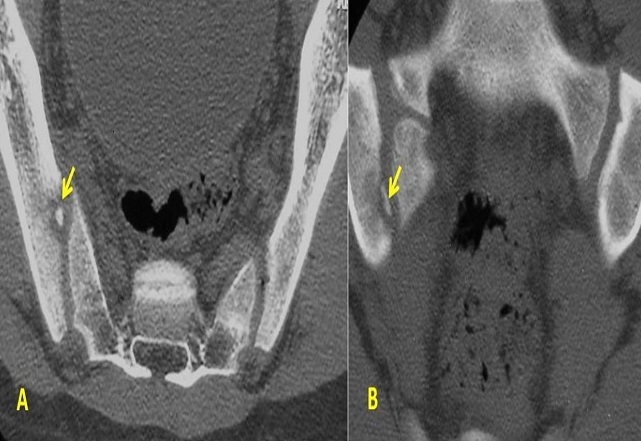
TDM du bassin. Coupes axiale et coronale des sacro-iliaques en fenêtre osseuse. Érosion localisée de la berge iliaque du pied de la sacroiliaque droite avec un petit séquestre en regard

**Figure 3:**
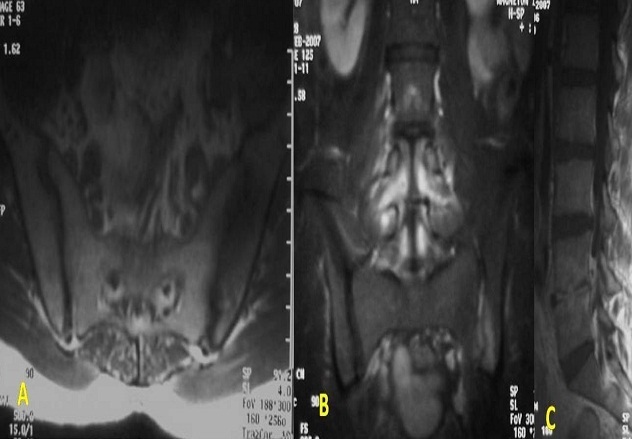
Sacroiliite gauche brucellienne sans collection des parties molles avec Spondylodiscite L4-L5 associée. A: Coupe IRM axiale T1: œdème médullaire diffus du versant iliaque de la sacroiliaque gauche en hyposignal T1 avec prise de contraste après Gado. B: coupe IRM coronale T1FS GADO: prise de contraste de l'interligne articulair. C: coupe sagittale T1FS GADO: prise de contraste du disque L4L5 avec épidurite antérieure

**Figure 4:**
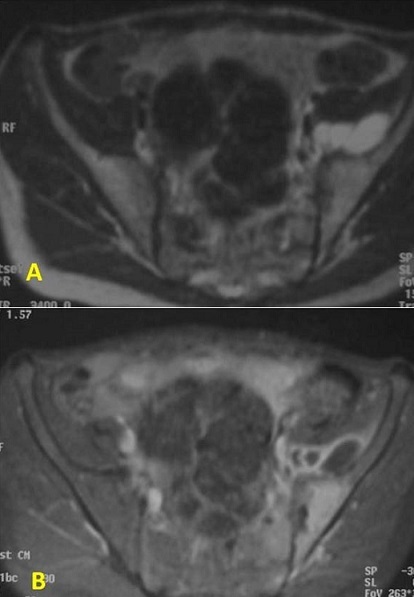
Coupes IRM axiales T2, T1FS GADO. Collection en hypersignal T2 avec prise de contraste périphérique en T1FS GADO. Œdème sous chondral et des parties molles périarticulaires avec une prise de contraste des berges sacrées et iliaques

Les radiographies standard du bassin étaient suggestives dans 13 cas en montrant des érosions floues des berges articulaires et un pseudo élargissement de l'interligne articulaire. Elles étaient normales dans les autres cas. La scintigraphie osseuse, réalisée dans 14 cas était positive dans 12 cas et avait montré une hyper fixation unique et localisée en regard de la sacro-iliaque (Figure 1, Figure 2, Figure 3, Figure 4).

La TDM, faite dans 13 cas, montrait un abcès des parties molles dans 8 cas et un séquestre osseux dans 2 cas. L'IRM, réalisée dans 8 cas, objectivait une infiltration des parties molles dans tous les cas et un abcès dans 3 cas. Le germe a été identifié dans 9 cas (3 cas de tuberculose, 3 cas de brucellose, 2 sacroiliites à pyogène et un cas de candidose). Cette identification était faite par biopsie dans 3 cas, hémocultures dans 2 cas, prélèvement à la porte d'entrée dans 1 cas et sérodiagnostic de Wright dans 3 cas. Le diagnostic de sacroiliite à pyogène était retenu sur des arguments cliniques et biologiques pour les autres cas.

## Discussion

Les SII surviennent préférentiellement chez l'enfant et l'adulte jeune. Les facteurs favorisants sont la toxicomanie, les infections cutanées et urogénitales, les traumatismes, la grossesse et le postpartum, les endocardites infectieuses et les néoplasies. L'inoculation bactérienne se fait le plus souvent par voie hématogène, mais peut aussi être due à un envahissement local [1]. En règle générale, la SII est unilatérale, même si des formes bilatérales ont pu être rapportées [2]. La symptomatologie unilatérale est un argument différentiel majeur pour écarter une spondylarthropathie. Dans notre série, l'atteinte était unilatérale dans tous les cas. La sémiologie fonctionnelle associe de manière diverse douleur, fièvre et boiterie. La douleur apparaît de manière brutale dans 75% des SI à pyogènes, et de manière progressive dans 75% des SI tuberculeuses [3]. Son rythme est inflammatoire et elle siège habituellement en pleine fesse, irradiant le plus souvent dans le membre inférieur dans le territoire sciatique, mais aussi dans la région lombaire, crurale, inguinale, voire dans la fosse iliaque, où elle peut simuler une douleur appendiculaire; dans notre série, la douleur était d'installation brutale dans 11 cas, son siège électif était la région fessière (10 cas).

La fièvre est en général présente. Elle est plus élevée dans les sacroiliites à germes banals que dans les sacroiliites tuberculeuses et peut néanmoins manquer surtout au cours des sacroiliites mélitococciques ou tuberculeuses. Son absence n’élimine en rien le diagnostic. Il n'y avait pas de fièvre dans 4 cas dans notre série.

Les examens biologiques montrent une augmentation quasi-constante de la vitesse de sédimentation globulaire, surtout au cours des sacroiliites à pyogènes. L'hyperleucocytose est inconstante et ne s'observe que dans les sacroiliites à germes banals. Une leucopénie (inférieure à 5000GB/mm3) peut être observée au cours de la brucellose, mais aussi parfois au cours des sacroiliites tuberculeuses [3], un syndrome inflammatoire biologique ainsi qu'une hyperleucocytose étaient retrouvés chez 11 patients de notre étude, la leucopénie était notée dans 4 cas uniquement.

La scintigraphie osseuse au technétium a une excellente sensibilité mais sa spécificité est faible. Elle est assez précocement positive et doit tenir compte de l'hyperfixation physiologique des sacro-iliaques vu leur turn-over élevé. Elle peut montrer une asymétrie de fixation évocatrice dès le deuxième jour; et peut néanmoins être prise en défaut jusqu'au quatrième jour au moins [4],dans notre étude, la scintigraphie réalisée dans 14 cas était positive dans 12 cas.

En imagerie, la radiographie standard peut être normale à un stade précoce (5 cas dans notre série). Les signes radiologiques apparaissent avec un délai de l'ordre de 15 jours pour les sacroiliites à pyogènes, et de 15 à 30 jours pour les sacroiliites mélitococciques et tuberculeuses [4].

La déminéralisation osseuse sous chondrale, responsable d'un aspect flou des berges, précède l’érosion géodique des surfaces articulaires, avec parfois constitution d'une image de séquestre. Les érosions sont sous la forme d'encoches de la lame osseuse sous chondrale profondes et floues pouvant réaliser l'aspect en « timbre de poste » des surfaces osseuses déterminent un élargissement de l'interligne articulaire. Ce sont les lésions les plus précoces et les plus caractéristiques.

Les séquestres osseux sont de petits fragments osseux intra articulaires au sein de l'ostéolyse des berges osseuses. Leur présence est une grande valeur diagnostique de l'origine infectieuse. L'hyperostose sous chondrale résultant d'un processus inflammatoire intra-osseux détermine une densification de l'os spongieux des berges articulaires prédominant sur le versant iliaque. Elle est étendue, floue et de faible densité. Elle se raccorde progressivement avec l'os spongieux sous jacent. L'ankylose articulaire est le stade ultime de l’évolution de la maladie. Elle se traduit par des ponts ou des tentatives de ponts osseux intra-articulaires aboutissant progressivement à la disparition de l'interligne. Les lésions élémentaires sus citées surviennent successivement et ne sont pas intriquées, ce qui est en faveur de l'origine infectieuse comme le soulignait Dihlmann: d'abord les érosions puis l'hyperostose et l'ankylose [5]. Le phénomène de vide articulaire n'a aucune spécificité diagnostique. Ces lésions sont difficiles à reconnaître vu les conditions souvent difficiles de réalisation des clichés chez ces patients hyperalgiques ( surprojection de gaz intestinaux résultant d'un iléus réflexe) [5].

Les méthodes d'investigation récentes, comme la TDM ou l'IRM, permettent de raccourcir le délai diagnostic et de prise en charge thérapeutique, elles présentent un intérêt considérable pour évaluer l'importance des lésions articulaires, réaliser une ponction guidée et effectuer un bilan d'extension locale.

La TDM permet la cartographie des lésions. Sa sensibilité est supérieure à celle de la radiographie standard [6, 7]. Elle montre l'élargissement de l'interligne articulaire, la déminéralisation juxta-articulaire, l'érosion de la surface corticale et de l'os sous-chondral et un stade tardif l'ankylose. Le premier signe de l'inflammation est la déminéralisation mais elle est difficile à évaluer [8].

Cette technique permet indiscutablement de reconnaître d'avantage de lésions érosives que la radiologie standard [5]. La TDM permet également de visualiser d’éventuels abcès endopelviens (8 cas dans notre série) ou la présence de séquestres intra-articulaires particulièrement difficiles à voir sur les radiographies standard [8]. Ces séquestres sont rares mais spécifiques de l'atteinte infectieuse (2 cas dans notre série: brucellose et tuberculose). L'atteinte des parties molles péri articulaires est un signe caractéristique de l'atteinte infectieuse. Elle est à type de tuméfaction, ‘dème, micro-abcès dans les formes débutantes et de véritables abcès dans les formes tardives et doit être recherchée sur les coupes axiales. Elle se traduit par des collections liquidiennes qui se rehaussent en périphérie après l'injection de produit de contraste. L’échographie peut être utile en cas de volumineuses collections qui sont bien limitées anéchogènes. Les voies de dissémination sont les muscles psoas iliaques et pyramidaux du bassin ainsi que la graisse située en arrière de l'articulation. Cet examen est également contributif pour guider les ponctions biopsie et les éventuels drainages de collection ainsi que le suivi post opératoire [7].

Le diagnostic radiologique de SII repose avant tout sur l'IRM qui est indiquée à la moindre suspicion du diagnostic. C'est l'examen le plus constamment et le plus précocement positif [9]. Elle est plus sensible que la radiographie standard, la TDM et la scintigraphie, avec une sensibilité de 85%. Sa spécificité est meilleure que la scintigraphie estimée à 90% en raison d'une meilleure résolution et l'atteinte des parties molles [9]. Cette technique est particulièrement utile chez la femme enceinte. L'IRM montre des anomalies de signal de l'os sous chondral et une prise de contraste intense de l'interligne articulaire et de la synoviale [4]. Les anomalies de signal de l'os sous chondral en rapport avec un ‘dème intramédullaire qui est le signe le plus précoce et le plus reproductible dans le diagnostic de sacroiliite; il se présente sous forme d'un hyposignal T1, hypersignal T2 avec saturation de la graisse et STIR avec un rehaussement après injection de Gadolinium [5].

Cette dernière séquence permet d'avoir un excellent contraste eau / graisse, de mieux détecter l’‘dème médullaire que la séquence T2 et de mieux détecter les phénomènes inflammatoires de l'articulation que sur la séquence T1 post Gadolinium [10]. L’‘dème médullaire est généralement diffus atteignant les deux versants de l'articulation [11]. La prise de contraste intra-articulaire de la synoviale et de la capsule articulaire après injection de Gadolinium est en rapport avec la synovite. L'atteinte du cartilage se traduit par des irrégularités de contours, variation nette et focale du signal de celui-ci qui perd son signal homogénéité. L'existence d'une infiltration ou d'abcès en dehors de la capsule articulaire et particulièrement dans les muscles adjacents (psoas-iliaque, gluteus) sont évocateurs de l'origine septique [9]. Les coupes en pondération T1 avec suppression du signal de la graisse sont particulièrement sensibles pour la détection de subtiles irrégularités des surfaces osseuses qui apparaissent en signal intermédiaire. Les érosions sont en hyposignal en T1 et en hypersignal T2 et STIR. La sclérose apparaît sous forme d'une zone en hyposignal ou asignal de l'os médullaire sur toutes les séquences et reste inchangée après injection de Gadolinium. Les ponts osseux trans-articulaires sont le premier signe de l'ankylose articulaire. Ils sont en hyposignal sur toutes les séquences. Au stade ultime l'articulation sacroiliaque se présente sous la forme d'une ligne de bas signal entourée par des dépôts graisseux.

Comme pour toute infection ostéoarticulaire, le diagnostic de certitude repose sur la mise en évidence du germe en cause. Ceci se fait par les hémocultures qui doivent être systématiques et qui sont positives dans 23 à 50% des cas et ont une grande valeur étiologique [3, 4], le prélèvement de la porte d'entrée, le dosage sérologique des anticorps, l'intradermoréaction et la recherche de BK dans les urines et les crachats. La ponction-biopsie au trocart permet de porter le diagnostic bactériologique et anatomopathologique. Cette technique, décrite par Chevrot et al. [12] et Vinceneux et al. [13, 14], est relativement simple et se réalise sous anesthésie locale et sous contrôle radiologique. Ce guidage radiologique permet de réduire la morbidité et d'augmenter la rentabilité de la ponction biopsie.

Les principales formes étiologiques des SII sont les pyogènes, la tuberculose et la brucellose.

**Sacroiliite à pyogènes:** Elles sont les plus fréquentes et d'installation souvent aigue, mais un tableau subaigu est possible. Le syndrome inflammatoire biologique est de règle avec augmentation de la vitesse de sédimentation (VS) et de la protéine C réactive (CRP), mais l'hyperleucocytose est inconstante, présente dans seulement 41% des cas dans la série de Feldman et al [3]. Les germes en cause sont: le staphylocoque doré (45-70% des cas), le streptocoque (9-10% des cas), notamment le streptocoque B dans le postpartum ou encore le pneumocoque, des bacilles Gram négatif qui peuvent être des entérobactéries banales, mais aussi le bacille pyocyanique chez le toxicomane intraveineux, et les salmonelles, rarement des germes anaérobies [3, 4, 15].

Le germe n'est pas identifié dans environ 1/3 des cas [16] (9 cas sur 19 dans notre série). Les hémocultures sont positives dans 23 à 50% des cas. On peut également recourir à un prélèvement d'un foyer infectieux à distance. Parfois, il est nécessaire de réaliser un prélèvement local qui peut être une ponction sous scanner en cas d'abcès des parties molles ou surtout une ponction'biopsie au trocart sous contrôle radiologique ou scannographique qui permet d’établir un diagnostic bactériologique dans 65 à 85% des cas (2 cas sur 3 dans notre série) [1]. Le traitement repose sur l'antibiothérapie selon les modalités habituelles recommandées dans les infections ostéoarticulaires [1]. Il est conseillé une bi-thérapie par voie veineuse adaptée au germe et poursuivie pendant 4 semaines en moyenne avec un relais per os qui sera pris ensuite pour une durée d'un mois au moins, voire plus en fonction de l’évolution.

**Les sacroiliites tuberculeuses:** L'articulation sacroiliaque est une localisation très classique de la tuberculose ostéoarticulaire. Les SI tuberculeuses représentent près de 10% des cas de tuberculoses ostéoarticulaires [17], elles touchent essentiellement l'adulte jeune.

Elle succède en général à une ostéite du sacrum qui envahit secondairement l'articulation. Habituellement, la sacroiliite tuberculeuse est unilatérale et d’évolution subaigue avec un début insidieux marqué par l'apparition d'une douleur habituellement inflammatoire. Une atteinte bilatérale est possible, retrouvée dans 6% des cas parmi 252 observations de la littérature [18]. Le délai diagnostique moyen est de quatre mois [19]. En conséquence, la radiographie est le plus souvent anormale lorsqu'on voit le patient, et les anomalies sont confirmées par la TDM et surtout l'IRM qui précisent l'atteinte des parties molles, notamment l'existence d'abcès froid [20]. Les lésions radiologiques de la SII sont d'installation très progressive [21].

L'atteinte est le plus souvent destructrice lors de sa découverte, elle est souvent associée à une autre localisation ostéoarticulaire, notamment à une spondylodiscite lombaire par l'intermédiaire d'un abcès du psoas [17] qui peut d'ailleurs en constituer le mode de présentation [20]. Les collections abcédées peuvent être également de siège fessier, dans le triangle de Scarpa ou la fosse iliaque. Elles peuvent être multiples, fistulisées et gigantesques à un stade évolué. Une localisation pleuro-pulmonaire peut être également associée. La vitesse de sédimentation est généralement modérément élevée. Le diagnostic nécessite le plus souvent un prélèvement local: ponction d'un abcès froid, et surtout ponction'biopsie de la sacroiliaque au trocart qui a l'avantage de pouvoir montrer rapidement un aspect histologique évocateur [20]: granulome épithélioïde et gigantocellulaire avec une nécrose caséeuse. Le rendement de la ponction biopsique sacro-iliaque dans le diagnostic de SI tuberculeuse, varie de 50 à 100% selon les séries [18]. Le traitement est initié avec une quadrithérapie antituberculeuse pour deux à trois mois, avec relais par l'association isoniazide et rifampicine. La durée totale du traitement recommandée est de 12 mois [19].

**Sacroiliites brucelliennes:** La brucellose est endémique dans certaines régions du monde, notamment dans les pays du bassin méditerranéen. L'atteinte de la sacroiliaque y est fréquente, représentant un quart à la moitié de l'ensemble des atteintes ostéoarticulaires de la brucellose, et touche surtout les adultes jeunes [22]. Elle est rare chez l'enfant. Elle survient habituellement à la phase aiguë de la maladie, sous la forme d'une sacroiliite unilatérale symptomatique. Le diagnostic repose d'abord sur la clinique: recherche d'un voyage en zone d'endémie ou d'une consommation d'un produit laitier non pasteurisé en dehors d'une zone d'endémie, recherche de signes évocateurs comme la fièvre sudoro-algique. Les hémocultures sont systématiques, mais la culture de Brucella est longue et difficile. Le diagnostic sérologique repose sur l'association du test standard de séroagglutination et du test de Coombs de la brucellose. Une sérologie franchement positive plaide fortement pour le diagnostic de sacroiliite mélitococcique [3]. Dans sa forme aiguë, la radiographie est normale. Si l'atteinte sacro-iliaque évolue sur une période plus longue, on peut voir apparaître un effacement des berges osseuses de la sacro-iliaque et un pseudoélargissement de l'interligne [23]. On peut avoir une association avec une arthrite de la hanche. Le traitement fait appel à une association d'antibiotiques, notamment les associations doxycycline et rifampicine, ou doxycycline et ciprofloxacine. L’évolution de la SI brucellienne est bonne avec une guérison des symptômes sous traitement [23].

### Diagnostic différentiel

Le diagnostic différentiel d'une sacroiliite est fonction de la technique d'imagerie utilisée. Sur la radiographie standard, l'arthrose peut être un diagnostic différentiel. Les érosions et l'ostéocondensation sous chondrale focale peuvent se voir dans l'arthrose mais siègent spécifiquement dans les zones de surcharge: partie antérieure du tiers moyen de l'articulation. Les pieds des sacroiliaques sont épargnés dans les atteintes dégénératives. Ces modifications osseuses sont symétriques dans les atteintes dégénératives, il s'y associe des remaniements dégénératifs pubiens [1].

Il existe également un développement d'ostéophytes antérieurs formant un pont osseux sur le versant ventral de l'articulation.

Il est utile de bien veiller à analyser séparément les tiers moyens/antérieurs et les portions postérieures et tout à fait inférieures. L'ostéose iliaque condensante se présente sous forme d'une image d'ostéocondensation triangulaire de la berge iliaque à limites nettes sans érosions articulaires [1] elle est facilement reconnue sur les radiographies ou le scanner et peut poser des problèmes d'interprétation en IRM [1]. Dans les sacroiliites inflammatoires, l'atteinte est bilatérale, les érosions, l'ostéocondensation et les ponts transarticulaires sont intriqués et panachés [5]. Elles surviennent n'importe où, c'est-à-dire que les lésions inflammatoires peuvent survenir dans le tiers antérieur/moyen des articulations, tout comme les formes arthrosiques mais également dans la partie la plus postérieure, et dans la partie tout à fait inférieure, pratiquement toujours respectées par les remaniements de type mécanique [5]. Les modifications sacro-iliaques de la spondylarthropathie ankylosante sont le plus souvent bilatérales et plus ou moins symétriques dans les formes chroniques mais elles sont très généralement asymétriques dans les formes débutantes (ainsi que de façon plus prolongée dans d'autres spondylarthropathies inflammatoires, arthrite réactionnelle et rhumatisme psoriasique par exemple).

En IRM, l’‘dème médullaire est focal ou multifocal pouvant prendre une distribution en patch qui prédomine, en général, sur le sacrum ou dans la région enthésique. L'inflammation ligamentaire enthésite est spécifique et se voit plus fréquemment à un stade tardif. Elle est représentée par un hyposignal en T1, un hypersignal en T2 avec saturation du signal de la graisse et un rehaussement en nappe des structures ligamentaires après injection de Gadolinium.

En IRM, l'aspect inflammatoire ou “oedémateux” d'une berge articulaire fera évoquer en première hypothèse une SI infectieuse ou inflammatoire, mais il peut aussi s'agir d'un oedème réactionnel à une fracture de contrainte ou à une tumeur [1]. Les lésions tumorales expansives des territoires sacro iliaques peuvent porter de façon non exceptionnelle sur les deux berges articulaires sans atteinte des surfaces cartilagineuses, par exemple en cas de sarcome, de lymphome, de myélome.

Les fractures de contrainte du sacrum en territoire juxta-articulaire sont caractérisées par des bandes d'impaction osseuse parallèles et juste en dedans des articulations, souvent bilatérales, parfois reliées par une fracture transversale. La radiographie est négative, elles sont suspectées par la scintigraphie et démontrées soit par tomodensitométrie, soit par IRM [24].

Les fractures de fatigue d'origine sportive sont des lésions fissuraires obliques en bas et en dedans. L'aspect en IRM est caractérisé en phase active par une plage de type oedémateux entourant une fine ligne vide de signal et en TDM par une bande de densification dont l'orientation est caractéristique. Les fractures de contrainte en cours de grossesse ou la lésion fissuraire présente l'aspect de fracture de fatigue [24].

## Conclusion

L'imagerie joue un rôle primordial dans le diagnostic précoce et l'orientation étiologique d'une sacroiliite infectieuse. Elle permet éventuellement l'identification du germe et confirme ainsi le diagnostic.
